# Resident Perceptions of Neighborhood Conditions, Food Access, Transportation Usage, and Obesity in a Rapidly Changing Central City

**DOI:** 10.3390/ijerph15061201

**Published:** 2018-06-07

**Authors:** Rayman Mohamed

**Affiliations:** Urban Studies and Planning, Wayne State University, Detroit, MI 48202, USA; ar7347@wayne.edu; Tel.: +1-313-303-6492

**Keywords:** obesity, fast food, litter, transportation

## Abstract

There is a lack of research on obesity that uses primary data and fine-grained information on neighborhoods. I use primary data for 367 participants in Detroit to examine neighborhood predictors of obesity. These data were supplemented with public data. I considered multilevel and spatial modeling, but the data lent itself best to ordinary least squares (OLS) regressions. I find that socioeconomic factors, the built environment, transportation usage, and perceptions of neighborhoods are important predictors of obesity. Importantly, litter is associated with higher levels of obesity. Planners can take measures to reduce litter and collaborate with other policy-makers to encourage less driving, though drawing direct lines of causality is complicated.

## 1. Introduction

Obesity continues to be a public health problem in the United States, with some 35 percent of Americans considered to be obese as measured by body mass index (BMI) [1]. Indeed, some 69 percent of Americans are considered overweight [2]. Several studies have linked socioeconomic status (SES) with high obesity rates in particular and poor health in general (and [3] for a more recent review; see [4] for an early review of this literature). For this reason, the study of obesity remains important.

In the last few decades, researchers have turned their attention to understanding behavioral and environmental factors that can affect obesity. Among these factors are broad categories of socioeconomic conditions, the built environment, transportation choices, and neighborhood conditions. While this literature has made progress, there are still gaps to be filled, particularly regarding the need to use primary data and fine-grained information on neighborhood conditions. This paper aims to address these issues in the literature by using primary data, including residents’ perceptions of their neighborhoods. The intention is to provide planners and policy-makers with guidance on initiatives that can reduce obesity.

## 2. Emerging Nonphysiological Concerns about Obesity

The continued prevalence of obesity has encouraged researchers to look at nonphysiological factors that can affect obesity. The literature has grown in the last decade. In this section, I distill the literature briefly into four categories that are pertinent to this research: socioeconomic status; the built environment, especially in terms of places that residents are likely to shop for food or eat unhealthful food; transportation modes; and neighborhood conditions. I acknowledge that this is a brief review of a large literature base and that categorizing the literature is not always straightforward.

### 2.1. Socioeconomic Factors

In general, the literature has found that higher levels of SES are inversely related to BMI [5]. Socioeconomic factors include race, marital status, living conditions, and education levels. For example, people with higher education have lower BMI [6], and higher-income Whites have lower BMI than lower-income Whites, though the effect is stronger for women [7]; (see also [8] for similar results from a study of adults in Germany). Similar results regarding race have been documented by other researchers [9,10,11,12]. Researchers such as Parsons et al. (1999) [13] suggest that disadvantaged adults tend to show higher BMI gains (Some research has found that older people are more obese [9], perhaps because they are less active. There is also literature on childhood obesity [2], but because this study was conducted on adults only, childhood obesity is not considered here.).

While the literature on how SES is associated with obesity is large, in many instances a lack of data has led researchers to rely on aggregate measures from large geographies rather than individual-level data obtained from surveys.

Over time, these SES variables have evolved to be control variables, as researchers turned their attention to understanding other nonphysiological factors that affect obesity.

### 2.2. The Built Environment

One area that has concerned scholars is the effect of the built environment on obesity [14,15,16]. Some of these studies include these references [17,18,19]. One element of the built environment is walkability. Research has shown that residents who live in highly walkable neighborhoods have lower obesity [20,21,22]. Similarly, in an Australian study, adults who accurately perceived their environments as highly walkable walked more and gained less weight, whereas those who perceived their environments as less walkable than they actually were walked less and gained more weight [23].

Scholars are concerned about walkability both for its intrinsic value in promoting good health and for its role in providing easy access to healthful foods, which is an important predictor of obesity (see [24] for a study of rural communities in the U.S.). In this regard, spatial access to healthful foods [22] and, conversely, spatial access to nonhealthful foods [25] have become important considerations. On the one hand, access to nonhealthful foods is often captured in terms of access to fast food (see [26,27]). For example, in focusing on middle-aged adults in low-income neighborhoods (the latter feature is particularly pertinent to the current research), Boone-Heinonen found that access to fast food, as measured by the number of establishments per 1000 residents within certain distances, increased fast food consumption. Convenience stores are also associated with higher levels of obesity [28]; such stores are likely to sell unhealthful foods.

On the other hand, several studies have reported that better access to full-service supermarkets is associated with lower weight or BMI, though the effect is small, and the studies use different definitions of access [28,29]. The implication of these papers is that full-service supermarkets are sources of healthful foods such as fruits and vegetables.

Access to healthful foods can be complicated by the above-mentioned SES factors. For example, studies cited earlier [9,10,11,12] note that African-Americans may be more likely than their White counterparts to be obese because they have less financial access to healthful foods.

The relationship between obesity and the built environment is not always consistent [30,31]. Indeed, some of the studies cited above highlight this point. For example, while Morland et al. (2006) [28] found that access to supermarkets reduces BMI, Boone-Heinonen (2011) [27] found no such relationship. Several issues make it difficult to draw universal conclusions, including difficulties in defining elements of the built environment and difficulties in defining how people navigate this environment [32,33,34] (see also the next subsection).

With regard to the definition of the built environment, one issue not acknowledged in the literature is how to define a supermarket—supermarkets range from large established companies (such as Kroger), where one can obtain healthful fruits and vegetables, to regional down-market chains (such as Glory Foods in the area examined in this study), which emphasize inexpensive processed and canned foods—nor are data in previous studies always clear about whether or how stores such as Family Dollar or Dollar General fit into this spectrum.

Another problem in defining the built environment is deciding on the location of features within neighborhoods. As in the case of SES, because of data limitations, some studies have relied on geographically expansive areas such as census tracts [22,28], counties [35], or states [36]. Other studies rely on densities of built features or population densities, for example, in the former case, densities of fast food restaurants in relatively large Forward Sortation Areas in Ontario [37] (though, to be sure, this study was concerned with cardiovascular events rather than obesity) and in the latter case, population densities over zip codes ([38] in a study of childhood obesity). Relying on densities over relatively large geographic areas means that residents are assumed to have the same access to these features regardless of whether they live immediately adjacent to them or many blocks away.

### 2.3. Transportation

Because the built environment is related to how people move within it, studies have examined the transportation habits of residents. Reliance on private, motorized transportation can lead to higher levels of obesity [21,39,40]. Dense, mixed-use developments can lead to less reliance on automobiles and, in turn, lower obesity [41]. Furthermore, areas with adequate public transit can encourage transit users to engage in more physical activity than non-transit users (e.g., [42,43]). Not surprisingly, residents in built environments that are more walkable have lower BMIs [44]. As many of these authors note, however, residents of these neighborhoods can be self-selecting, as healthier people inclined to more physical activity are more likely to live in these places (e.g., [41]).

However, dense, mixed-use neighborhoods are not the only determinant of transportation choices. Because of the location of jobs, people may have to commute long distances. Thus, studies that have examined obesity and transportation frequently measure vehicle miles travelled (VMT), automobile dependency, commuting time, or commuting distance. These studies find that increased VMT (see, e.g., [35]), automobile dependency [45], and commuting time are associated with higher BMI [45,46].

As in the case of the built environment, several data-related issues need to be considered or acknowledged. Some studies consider network distances along roads, while others consider straight-line distances. Both approaches can be justified, but ultimately their accuracy depends on whether residents rely on cars for navigation (those who do are more likely to use network distances to navigate) or walk or bike to many destinations. The transportation modes of walking and biking are further complicated by the conditions of sidewalks and the availability of unmapped routes such as those that traverse vacant land, much of which exists in the study area. These are important considerations since research has shown that modes of transportation affect obesity (e.g., [42]).

There is also reason to believe that everyday transportation habits matter. It appears that there are no studies that combine data on the built environment with people’s everyday travels. Living in walkable environments with access to public transportation may matter less for predicting obesity if getting to work requires automobile travel. Indeed, some people may prefer to use their cars for destinations that others might consider to be accessible by walking, which suggests that it is important to consider both the built environment and everyday traveling habits.

### 2.4. Residents’ Perceptions of Their Neighborhoods

While the literature has grown to address the areas discussed above, there is less research on how residents’ fine-grained perceptions of their neighborhoods affect obesity rates. For example, while there are studies on the presence of sidewalks on obesity (e.g., [47]), I can find no studies on perceptions of litter or blight, two conditions that can reasonably be expected to affect walking and in turn obesity. Thus, dense, walkable neighborhoods that are unkempt or contain numerous abandoned properties can be expected to discourage walking even though they are dense.

There is, however, literature on crime and obesity. Generally, the mechanism through which crime is thought to affect obesity is that it discourages physical activity [48,49]. A review of this literature by Yu and Lipper (2016) [50] highlights the important association between violent crime and higher levels of obesity, and the authors suggest that violent crime may be especially discouraging to physical activity. While crime rates suffer from some of the same data limitations as data on the built environment, such as using crime densities over relatively large areas, recent advances in geocoding crime incidents have allowed researchers to obtain crime data at fine levels of geography. Aside from crime, however, there is a lack of literature on fine-grained neighborhood characteristics.

### 2.5. The Use of Self-Reported BMI

A shortcoming in some of the literature reviewed is that lack of resources has led researchers to conduct some studies through telephone surveys. Examples of these studies include [9,51,52]. The disadvantage of this process is that participants might give inaccurate information about their weight and height, the two factors that go into calculating obesity.

### 2.6. Differences from Previous Studies and Contributions to the Literature

This paper aims to add to our understanding of how the built environment affects BMI by addressing some of challenges that other research has faced. While I cannot address all of the challenges, I attempt to address some of them by (1) using consistent, in-person protocols to obtain weights and checking the heights of participants ourselves, thereby avoiding self-reported BMIs; (2) obtaining socio-economic measures through surveys of our participants rather than relying on aggregate census tract or county measures, which themselves are too large to reliably attribute to individuals; (3) geocoding addresses of participants rather than placing them at the centroid of census tracts or counties; (4) obtaining information on the location of supermarkets, dollar stores, gas stations, etc., from publicly available data and verifying these with local knowledge; (5) geocoding the location of these features in the built environment rather than using measures of density at the tract or county level (e.g., the number of supermarkets in the tract or county); (6) simultaneously collecting information on people’s transportation habits rather than relying on aggregate geographic-based transportation measures; and (7) obtaining information on participants’ perceptions of their own neighborhood, particularly their perceptions of litter and blight. As far as I am aware, this is the first study to combine these data simultaneously.

## 3. Data and Approach to Analysis

**Figure 1:**
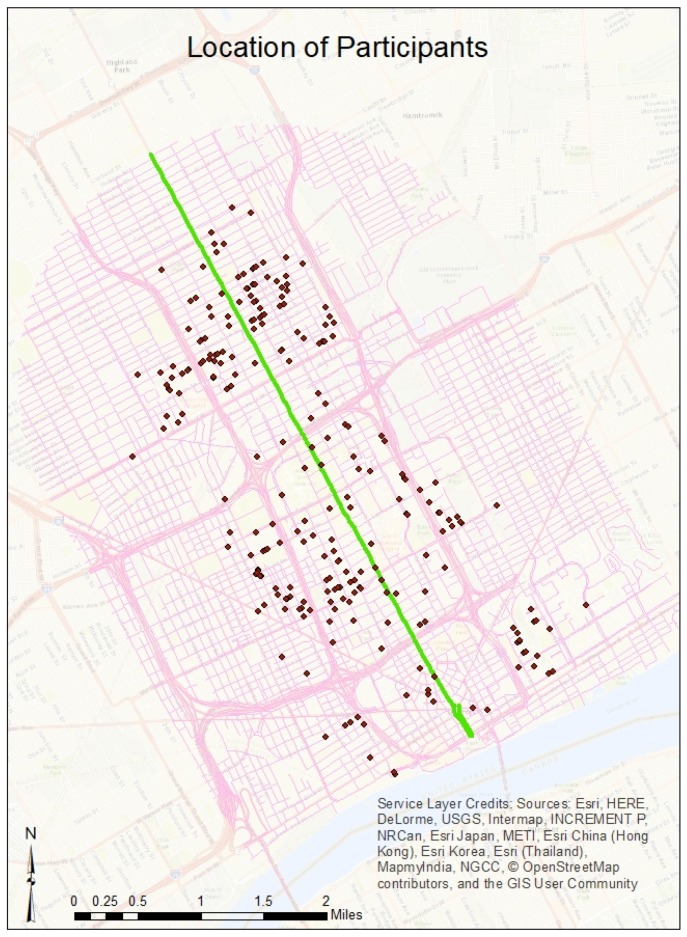
The locations of respondents to the survey.

The data were obtained from a combination of sources: a survey of 367 participants in and around the revitalizing Downtown, Midtown, and New Center of Detroit (see Figure 1); a publicly available portal maintained by the City of Detroit; publicly available data from the City of Detroit; the City’s Health Department; and publicly available listings of relevant data on the built environment. Data were collected from participants who live approximately one mile around a newly planned streetcar called the Q-Line on a rapidly revitalizing stretch of Woodward Avenue, the main thoroughfare in Detroit (At the time of writing, the building of the Q-Line has been completed.). I chose this area for study because many areas immediately adjacent to the Q-Line (and Woodward Avenue) are undergoing rapid change, though a few blocks in some neighborhoods are still experiencing blight (This research is part of a longer-term plan to study the health and transportation changes associated with the Q-Line and the rapidly changing areas that it covers.).

Institutional Review Board approval was granted to perform the surveys. Participants were selected by sending notices to randomly selected addresses. Those interested in the survey used a phone number on the notice to call for an appointment. The appointment was scheduled on the campus of Wayne State University and the surveys were administered by a research assistant and the author. Only one adult per household was permitted to take part in the survey and this criterion was verified by checking government-issued driver’s licenses or ID cards. Participants’ weights and heights were recorded to calculate BMI. The survey covered questions as further discussed below.

### 3.1. Socioeconomic Characteristics

Drawing on the literature discussed earlier, I collected data on the following socioeconomic characteristics: sex, marital status, whether living alone or not, household income, educational status (measured in terms of having an associate’s degree or less), and self-declared health status (For simplicity, I consider self-declared health conditions to be an SES variable.). Although the literature on age is not conclusive, I included it as an additional independent control variable.

### 3.2. The Built Environment

Data on the built environment were collected from various public sources. Data on the locations of liquor stores, gas stations, supermarkets (I categorized Eastern Market, a large historic public market, as a supermarket), and dollar stores were obtained through a mixed process using local knowledge of the study area, data from the open data portal, and Google searches. The locations of fast food restaurants were obtained from Detroit’s Department of Public Health (Data on the locations of fast food restaurants were kindly contributed by Alex Hill of the Detroit Public Health Department.).

Using the home addresses of participants, I determined distances to features of the built environment noted above. I chose straight-line distances instead of street network distances because of the high number of vacant lots in Detroit and the deteriorated condition of many sidewalks. The abundance of vacant lots means that while people use street networks to drive to destinations, there are no guarantees that they use them for biking or walking. The poor condition of sidewalks in some areas further suggests that people might not use established networks to bike or walk, which implies that network distances might be unreliable.

I distinguish between gas stations and dollar stores on the one hand and supermarkets on the other hand because the former sell primarily prepared foods and snacks, while the latter also sell fruits, vegetables, and meat. In any event, I acknowledge the fluid nature of what gas stations, dollar stores, and supermarkets sell in terms of healthful and unhealthful foods. To help control for this, I combined gas stations and dollar stores into a single category in the analyses because both are likely to focus on unhealthy foods and neither sell alcohol. I considered liquor stores as a separate variable because their main line of business is selling alcohol, which I posit can be associated with an unhealthful lifestyle.

All data on the built environment were collected for a 2 mile distance around the QLine to allow for the fact that participants—even though they live within one mile of the QLine—can travel outwards to go to these places.

### 3.3. Transportation Choices

Transportation choices were determined by providing participants with a transportation usage log, in which they were asked to record daily the number of automobile trips, public bus trips, and bicycle and walking trips that lasted more than 10 min. Participants kept these logs for one week. Upon returning the logs, they received $75 for their participation (Some of our participants were provided with Fitbit devices. These participants were compensated with $100 instead of $75. This paper does not include any analysis of Fitbit data.). Data were collected between September 2016 and March 2017. Because some of the data collected are related to transportation use, such data should ideally be collected during the spring and fall months (after school has started), when transportation patterns are more stable. However, I lacked capacity to collect data within a brief period; hence, I decided to collect data over a longer period. Even so, the process was organized so that data were collected after the school year started in the fall, and no data were collected during Thanksgiving week or between approximately 20 December 2016 and 5 January 2016. I included a dummy variable in the regression to distinguish between data collected before 16 November 2016 and after 31 March 2017 on the one hand and data collected between 16 November 2016 and 31 March 2017 on the other hand. This is represented in the tables as Fall/Spring.

### 3.4. Neighborhood Conditions

We asked participants questions about their perceptions of litter and blight in their neighborhoods. Data on another neighborhood-related characteristic—crime—were obtained from the City of Detroit’s public data portal. Two further points are warranted with respect to the crime data:

- I mapped only felonies when considering crime rates. I use a liberal definition of felonies to include incidents that included crimes ranging from property damage to murder.
- I population-normalized crime rates at the block group level. I would prefer to normalize at the block level, as a smaller geographic area would better capture the effects of crime on physical activity. However, census population estimates at the block level are unreliable.

As an additional neighborhood variable, I obtained a walkability measure for each address from Walk Score (data provided by Redfin Real Estate in Detroit). Table 1 shows descriptive statistics for all data.

### 3.5. Approach to Data Analyses

Public health data are frequently examined using multilevel models (MLM; see, e.g., [53]) because participants are organized in households or nested neighborhoods. However, because my sample contains only one person per household—and for three other reasons summarized by Shah (2017)—I do not employ MLMs. These three reasons are as follows: (1) Consistently defining neighborhoods can be difficult because neighborhoods can vary in scale, shape, and size [54,55,56] (One broad definition of neighborhoods is Downtown, Midtown, New Center, and North End. However, these distinctions hold only in the north–south direction immediately parallel to Woodward Avenue. Just a few blocks east–west from Woodward Avenue, circumstances can change dramatically. For example, moving east–west in the North End, one sees that just one or two blocks east or west of Woodward Avenue, the neighborhoods are very different socioeconomically than those right next to Woodward Avenue; thus, North End is too broad a geography to classify as a neighborhood. The same holds true for all the other broadly defined neighborhoods, with the possible exception of Downtown. Another possible definition of the neighborhood is the block level, but, as noted earlier, some sociodemographic data are not reliable at the block level. Block-level definitions are further complicated because neighborhood phenomena are not adequately captured at the block level. For example, crime that takes place in one block may have spillover effects on several surrounding blocks); (2) MLMs treat neighborhoods or households as if they were independent units without spatial interaction, which ignores spatial peer effects on behavior [57]; (3) As Shah (2017, 109) notes, while there might be clustering by participants, this clustering may not mean that participants belong to the same group; rather, they may simply be in the same geographic area. In fact, these last two possibilities suggest the presence of spatial spillover effects.

These spillover effects suggest another approach to analyzing the data: spatial regression models (see, e.g., [57]). An example of this approach can be found in [58]. However, an analysis of the data used in this research did not reveal spatial clustering of BMIs: tests for *Moran’s I*, a measure of spatial correlation, were not statistically significant. I also explored the possibility of using hierarchical regression models because there is reason to believe that socioeconomic factors, built-environment factors, and transportation habits can be entered sequentially into a regression model. However, hierarchical regression produced no better results than the ordinary least squares (OLS) regression. The absence of spatial spillover effects is consistent with other research that has not observed such effects (Shah, 2017). For this reason and the reasons outlined in the previous paragraph, I decided to use ordinary least squares regressions.

## 4. Results

Table 2 shows the results of the OLS model. Some socioeconomic variables are associated with obesity, consistent with most other research. Those that are significant at the 5 percent level or better are being female, being Black, being married, and living alone. The first two variables are associated with increases in obesity: females have BMIs that are about 6.9 percent higher than those of males, and Blacks have BMIs that are about 8.8 percent higher than those of people of other races. The second two variables are associated with decreases in obesity. Being married reduces one’s BMI by 8.2 percent and living alone reduces one’s BMI by 5.4 percent.

Having an associate’s degree or less also increases BMI, this time by about 5.1 percent. However, this result is significant at the 10 percent level.

Not surprisingly, those who describe their health as very good or excellent have lower BMIs than those who describe their health as poor, fair, or good; their BMI is lower by about ten percent (significant at better than the 1 percent level).

Important elements of the built environment are associated with higher obesity. A 10 percent increase in distance away from the closest fast food restaurant leads to about a 0.4 percent decrease in obesity. Conversely, a 10 percent increase in distance away from the closest supermarket leads to about a 0.2 percent increase in obesity. Both results are significant at the 5 percent level.

In terms of transportation use, each additional automobile trip per week results in about a 0.3 percent increase in obesity, a result that is significant at the 5 percent level. The other transportation variables are not statistically significant.

Among neighborhood conditions, litter clearly emerges as an issue. Respondents who considered their neighborhoods to have a moderate to extreme litter problem had obesity levels that were about ten percent higher than the obesity levels of those who did not consider their neighborhoods to have a serious litter problem (significant at the 1 percent level). However, perceptions of blight, crime rates, and walkability did not have any effect on participants’ BMI, and the final control variable to account for colder months was not statistically significant either.

## 5. Discussion and Conclusions

The results point to the importance of socioeconomic factors, the built environment, transportation choices, and residents’ perceptions of their neighborhoods as important predictors of obesity. As far as I am aware, this is the first research to bring together all of these issues in a study of obesity. I believe it is also the first study to use such an extensive collection of primary data.

### 5.1. Socioeconomic Factors

Although investigating the effects of SES was not a central objective of this research, some comments on these factors are warranted. In general, the results are broadly consistent with research that has found that females and Blacks are more likely to be obese, while being married is associated with less obesity. Similarly, the results for level of education and health status are not surprising (It is not surprising that age is not statistically significant. Another approach to the analyses would have been to use age cohorts, such as 17–29, 30–39, etc., but such an approach might have used up too many degrees of freedom in the data.).

I cannot explain the contradictory result that living alone is associated with less obesity. It may be because living alone does not mean that residents are not married; it is possible that many married residents may live alone in the study area. In any event, it is surprising that living alone is associated with lower levels of obesity because there is reason to believe that those who live alone are less likely to lead lifestyles amenable to lower obesity [59].

### 5.2. The Built Environment

In terms of the built environment, two independent variables emerge as important: distance to fast food restaurants and distance to supermarkets. These results add to a growing body of literature about the importance of proximity to healthful foods [22,25]. Being near fast food restaurants can have deleterious effects on weight. The fact that the result for full-service supermarkets is statistically significant in the presence of other stores that served as mini-supermarkets (dollar stores, liquor stores, and gas stations) emphasizes the importance of the former in lowering obesity rates.

The connections identified here are likely causal rather than indirect effects; there are good theoretical reasons to believe that easier access to healthful foods and—vice versa—more difficult access to healthful foods influence what one eats, especially after accounting for the socioeconomic variables included in these analyses.

It is not possible to move fast food restaurants out of the neighborhood or to introduce more supermarkets to the area, nor do policy-makers who shape the built environment have much influence over food prices. Nonetheless, when combined with other results from this study, these results offer support for educational and other activities aimed at influencing where people shop and what they eat. The results could be related to neighborhood conditions, as I discuss later.

### 5.3. Transportation Choices

A variable such as the number of automobile trips is an easy objective measure for participants to record. Not surprisingly, more automobile trips lead to higher levels of obesity (see, e.g., [40]). The following factors may influence why the other forms of mobility were not statistically significant:

- The number of bus trips is complicated by the fact that participants may walk long distances from the final bus stop to the eventual destination. On the other hand, participants reported that it took them, on average, only 3.2 min (standard deviation of 2.8 min) to walk from their home to the nearest bus stop, with a maximum time of only eight minutes (these figures do not appear in the tables). These data reflect the high density of bus stops in the study area.
- The number of bicycle trips is complicated by the fact that participants may combine bicycle and bus trips or may take bike trips that are too short to have an impact on obesity.
- The number of walking trips is complicated by several factors. For example, our debriefing with participants revealed that it was difficult to count the number of walking trips that lasted more than 10 min (our instruction to participants) because it was difficult to ascertain whether some trips—such as running an errand—lasted that long.

I thus believe that more accurate ways of tracking bicycle and walking trips might lead to results that are consistent with expectations; that is, I would likely find that more bicycle and walking trips lead to lower levels of obesity.

### 5.4. Neighborhood Conditions

One clear result is the association of litter with obesity. While litter itself may be a direct deterrent to people’s willingness to be active in their neighborhoods, it is likely to be a broader indication of other conditions that encourage less physical activity, such as vacant land, overgrown brush, or deteriorated sidewalks. There could of course be complicated relationships between socioeconomic factors and obesity, but even if litter is a proxy for variables such as these, it is easier to address these conditions than to persuade people to eat more healthily or to move fast food restaurants or supermarkets. This is an appealing result for policy-makers because it is reasonable to assume that removing litter and maintaining a litter-free environment is less expensive than other approaches.

Another consideration is the possibility of reverse causality or simultaneity between obesity and litter. It might be that local governments are less timely in removing litter and addressing other visible signs of deterioration in areas where people have less political influence. However, even if this is the case, again, addressing problems of litter is relatively easy for local governments to do.

I expected blight to be a statistically significant variable as well. However, unlike litter, blight may be more difficult for participants to identify consistently. For instance, different participants may have different perceptions of what constitutes blight. On the other hand, perceptions of litter are likely to be more reliably reported by participants.

It is important to note that neighborhood variables are not directly responsible for high obesity rates. These variables may instead act as deterrents to physical activity. For example, high crime rates (though not statistically significant in this research) may make it unsafe to exercise [60,61], which may be responsible for higher levels of obesity (see [50] for a review of crime and obesity).

### 5.5. Implications for Planning

The results have implications for planning. While—from a planning perspective—little can be done about various sociodemographic characteristics or the location of fast food restaurants or supermarkets, there are three areas where planners can intervene. First, more litter is associated with higher levels of obesity. While the pathways from litter to obesity can be complicated, this factor nonetheless suggests an intervention that is relatively inexpensive and that may have benefits beyond obesity, such as increasing property values and attracting new residents to cities.

Second, while zoning and other regulations can influence the location of fast food restaurants and supermarkets, in the end these decisions are made by the private sector. Nonetheless, since access to food clearly influences levels of obesity, planners can collaborate with other health policy professionals to promote healthful eating.

Third, while active modes of transportation were not statistically significant, the fact that automobile usage is associated with higher levels of obesity provides planners with additional public health arguments to reduce automobile dependency by encouraging mixed-use development and more public transportation.

## Figures and Tables

**Table 1:** Descriptive statistics of variables of interest.

Variable	Minimum	Maximum	Mean	Standard Deviation
Natural log BMI	2.79	3.93	3.34	0.21
Socioeconomic characteristics				
Age	17	88	53.31	15.35
Female			0.57	0.50
Black			0.82	0.38
Married			0.13	0.34
Live alone			0.65	0.48
Household income	0	350,000	26,782	37,704
Associate’s degree or less			0.75	0.43
Health very good or excellent			0.35	0.48
Built environment				
Natural log distance to fast food restaurants	2.57	6.78	5.80	0.72
Natural log distance to liquor stores	3.18	6.79	5.58	0.77
Natural log distance to supermarkets	1.74	7.41	6.17	0.99
Natural log distance to dollar stores and gas stations	3.76	7.13	6.03	0.61
Transportation use				
Automobile trips per week	0	53	12.09	11.53
Bus trips per week	0	64	3.65	6.96
Bicycle trips per week	0	20	0.85	2.69
Walking trips per week	0	60	5.66	7.38
Neighborhood conditions				
Moderately to very concerned about litter in their neighborhood			0.77	0.42
Moderately to very concerned about blight in their neighborhood			0.73	0.45
Crime rate per 100 residents, Block Group	1.86	61.80	10.04	9.61
WalkScore	42.00	100.00	77.30	14.07
Additional control variable				
Fall/Spring			0.43	0.50

BMI: body mass index.

**Table 2:** Results from the ordinary least squares (OLS) regression.

Variable	Coefficient	Standard Error	Level of Significance
Constant	3.212	0.180	***
Socioeconomic characteristics			
Age	0.001	0.001	
Female	0.067	0.022	***
Black	0.084	0.034	**
Married	−0.086	0.037	**
Live alone	−0.056	0.028	**
Household income	2.651 × 10^−7^	0.000	
Associate’s degree or less	0.050	0.031	*
Health very good or excellent	−0.107	0.024	***
Built environment			
Natural log distance to fast food restaurants	−0.042	0.018	**
Natural log distance to liquor stores	0.021	0.019	
Natural log distance to supermarkets	0.023	0.011	**
Natural log distance to dollar stores and gas stations	−0.007	0.018	
Transportation use			
Automobile trips per week	0.002	0.001	**
Bus trips per week	−0.001	0.002	
Bicycle trips per week	−0.003	0.004	
Walking trips per week	−0.001	0.001	
Neighborhood conditions			
Moderately to very concerned about litter in their neighborhood	0.097	0.035	***
Moderately to very concerned about blight in their neighborhood	−0.034	0.032	
Crime rate per 100 residents, Block Group	−0.001	0.001	
WalkScore	0.000	0.001	
Additional control variable			
Fall/Spring	−0.001	0.022	

*N* = 339; Adjusted *r*^2^ = 0.21; *** Significant at the 1 percent level; ** Significant at the 5 percent level; * Significant at the 10 percent level.
